# Differences in Energy Balance-Related Behaviours in European Preschool Children: The ToyBox-Study

**DOI:** 10.1371/journal.pone.0118303

**Published:** 2015-03-18

**Authors:** Marieke De Craemer, Mina Lateva, Violeta Iotova, Ellen De Decker, Maïté Verloigne, Ilse De Bourdeaudhuij, Odysseas Androutsos, Piotr Socha, Zbigniew Kulaga, Luis Moreno, Berthold Koletzko, Yannis Manios, Greet Cardon, the ToyBox-study group

**Affiliations:** 1 Ghent University, Ghent, Belgium; 2 Medical University Varna, Varna, Bulgaria; 3 Research Foundation Flanders, Brussels, Belgium; 4 Harokopio University, Athens, Greece; 5 Children’s Memorial Health Institute, Warsaw, Poland; 6 University of Zaragoza, Zaragoza, Spain; 7 University of Munich Medical Centre, Munich, Germany; University of Bath, UNITED KINGDOM

## Abstract

**Background:**

The aim of the current study was to compare levels of energy balance-related behaviours (physical activity, sedentary behaviour, and dietary behaviours (more specifically water consumption, sugar-sweetened beverage consumption and unhealthy snacking)) in four- to six-year-old preschoolers from six European countries (Belgium, Bulgaria, Germany, Greece, Poland, and Spain) within the ToyBox cross-sectional study.

**Methods:**

A sample of 4,045 preschoolers (4.77 ± 0.43 years; 52.2% boys) had valid physical activity data (steps per day), parents of 8,117 preschoolers (4.78 ± 0.46 years; 53.0% boys) completed a parental questionnaire with questions on sedentary behaviours (television viewing, computer use, and quiet play), and parents of 7,244 preschoolers (4.77 ± 0.44 years; 52.0% boys) completed a food frequency questionnaire with questions on water consumption, sugar-sweetened beverage consumption and unhealthy snacking.

**Results:**

The highest levels of physical activity were found in Spain (12,669 steps/day on weekdays), while the lowest levels were found in Bulgaria and Greece (9,777 and 9,656 steps/day on weekdays, respectively). German preschoolers spent the least amount of time in television viewing (43.3 min/day on weekdays), while Greek preschoolers spent the most time in television viewing (88.5 min/day on weekdays). A considerable amount of time was spent in quiet play in all countries, with the highest levels in Poland (104.9 min/day on weekdays), and the lowest levels in Spain (60.4 min/day on weekdays). Belgian, German, and Polish preschoolers had the lowest intakes of water and the highest intakes of sugar-sweetened beverages. The intake of snacks was the highest in Belgian preschoolers (73.1 g/day) and the lowest in Greek preschoolers (53.3 g/day).

**Conclusions:**

Across six European countries, differences in preschoolers’ energy balance-related behaviours were found. Future interventions should target European preschoolers’ energy balance-related behaviours simultaneously, but should apply country-specific adaptations.

## Introduction

The high prevalence of overweight and obesity is a worldwide health problem, and is already manifest in preschool children [1]. Childhood overweight is associated with serious health problems, the risk of premature illness and early death later in life [1]. In developed countries, the prevalence of overweight and obesity among children under the age of five years increased from 7.9% in 1990 to 11.7% in 2010 with an expected prevalence of 14.1% in 2020 [1]. In Europe, the prevalence of overweight in preschool boys ranges from 10% in Belgium and Germany to 30% in Spain. In preschool girls, it ranges from 8% in Germany to 30% in Spain [2]. Changes in lifestyle behaviour are likely to be the main cause of the increase in overweight and obesity, rather than changes in biologic or genetic factors [3]. Weight gain is determined by the cumulative effect of low levels of physical activity (PA), high levels of sedentary behaviour (SB), and unhealthy dietary behaviours, also referred to as energy balance-related behaviours (EBRBs) [4]. Focusing on several EBRBs could be more effective in overweight and obesity prevention in preschoolers, since focusing on one single EBRB as a universal causal factor of overweight and obesity is not ideal because it is the co-existence and interaction of these specific EBRBs that results in preschoolers’ weight gain [4, 5]. Furthermore, establishing healthy lifestyle behaviours—such as more PA, less SB, and healthy dietary behaviours—is already important at an early age, since these healthy EBRBs track into later life [6–9], and even into adulthood [10, 11].

For PA, SB and dietary behaviours, guidelines have been developed for children at preschool age. The most recent PA guidelines for preschool children suggest that preschoolers (one- to five-year-olds) should be physically active every day for at least three hours (180 minutes) at any intensity, spread throughout the day [12–15]. This guideline corresponds to taking 11,500 steps/day [16].Next to the suggestion to limit prolonged periods of sitting [17–19], SB guidelines also include specific recommendations for the amount of screen time per day, because screen time is the most common SB in preschool children, and is therefore frequently used as a proxy marker of overall SB in preschoolers [12, 20]. Recent guidelines recommend that preschool children (one- to five-year-olds) should limit watching television (TV) and the use of other electronic media—like computer, DVDs and other electronic games—to less than one hour per day [17]. However, overall sedentary time is not made up of screen time alone, so it is important to measure preschoolers’ time spent in quiet play (e.g., drawing, tinkering, puzzling, looking into books) as well, since quiet play is categorized as productive SB [12]. Food-based dietary guidelines (FBDG) for preschool children only exist on a national basis. The FBDG used in the present study are based upon the nutrient recommendations of the Belgian Health Council and the World Health Organization, combined with data on habitual dietary intake in the Belgian population [21]. These FBDG are very similar to dietary guidelines in other countries, making these guidelines applicable for a European population of preschoolers [22]. The Belgian FBDG suggests limiting the consumption of sugar-sweetened beverages (SSBs) and unhealthy snacks, since these kinds of beverages and foods are strictly not necessary in a balanced diet (e.g., soft drinks, candy, potato chips). Furthermore, preschool children should consume 500 ml to 1,000 ml water per day [23].

To date, there are not many studies investigating several EBRBs simultaneously in preschool children. Only one study simultaneously compared EBRBs in preschoolers across six European countries (Belgium, Bulgaria, Germany, Greece, Poland, and Spain) by executing a pooled analysis of six European studies [2]. However, since data from six different studies were used, results could not be compared due to differences in the variables of interest, in sample sizes, and differences in measurement methods [2]. Large cross-European studies examining preschoolers’ engagement in EBRBs, assessed by the same measurement methods, are currently lacking. The present study presents data on four- to six-year-old preschoolers’ EBRBs from six European countries, assessed with the same measurement methods [24]. Preschoolers’ EBRBs were examined across six European countries to expose local and cultural differences and similarities, which could serve as a base for the development of future interventions targeting overweight and obesity in preschool children, as the interaction and co-existence of these three EBRBs (i.e., PA, SB and dietary behaviour) has an influence on preschoolers’ weight status [4]. These data could inform policy makers and research institutions on which behaviours to target in which countries. We specifically focused on four- to six-year-old children since this is the critical period for the adiposity rebound. At this age, body adipose tissue reaches a post infancy low point (typically at the age of four to six years) [25].

## Methods

### Participants

All subjects in the present study participated in the ToyBox cross-sectional study, with the aim to collect information on the prevalence of overweight and obesity, and on EBRBs and their correlates across six European countries: Belgium, Bulgaria, Germany, Greece, Poland, and Spain. Per country, kindergartens were selected in the provinces of West- and East-Flanders in Belgium, Varna in Bulgaria, Bavaria in Germany, Attica in Greece, Warsaw and surroundings in Poland, and Zaragoza in Spain. Within each of these provinces, kindergartens were recruited from different socio-demographic backgrounds. In all countries, lists of all municipalities that exist within the selected provinces were created and information on the socio-economic status (SES) variables was provided (years of education for the population of 25–55 years (cut-off: >14 years of education) or annual income (quantitative variable)). Tertiles were created, based on the selected SES variables, and each country randomly selected five municipalities per SES status (thus, five municipalities for low SES, five for medium SES, and five for high SES). Then, kindergartens within these randomly chosen municipalities were randomly selected (with the exclusion of the lowest 20% of the kindergartens with the smallest number of pupils). Power analyses were performed before the start of the study, and were based on a previous school-based intervention study [26]. The analyses showed that a sample size of at least 800 preschool children per country would be sufficient to detect changes in EBRBs and their determinants during future follow-up measurements. To account for possible drop-out, each country had to recruit an initial number of 1,100 preschoolers, which would make a total sample of 6,600 preschool children across all six countries. Particularly in Greece, over sampling occurred due to the transition from kindergarten to primary school at the age of five in Greece, which means that these children would be located in other institutions in Greece during future follow-up measurements. Data collection occurred between March and June 2012. Furthermore, preschoolers’ parents/caregivers provided written informed consent before being enrolled in the study (only in Belgium, parents/caregivers provided passive informed consent).

### Ethics statement

This study was included in the approval of the ToyBox-study by Ethical Committees in all six European countries, in line with national regulations (i.e., the Ethical Committee of Ghent University Hospital (Belgium), Committee for the Ethics of the Scientific Studies (KENI) at the Medical University of Varna (Bulgaria), Ethikkommission der Ludwig- Maximilians-Universität München (Germany), the Ethics Committee of Harokopio University of Athens (Greece), Ethical Committee of Children’s Memorial Health Institute (Poland), and CEICA (Comité Ético de Investigación Clínica de Aragón (Spain)).

### Procedure

Measurements were conducted according to standardized protocols. The procedure of data collection, data deposition and data reporting was standardized and harmonized within the ToyBox-study. Preschoolers between 3.5 and 5.5 years old were fitted with a motion sensor to assess their PA levels, and they received two parental questionnaires (core questionnaire and food frequency questionnaire (FFQ)) in a closed envelope to take home for completion by one of the parents/caregivers.

#### Physical activity

PA was assessed by means of steps per day using Omron Walking Style Pro pedometers (HJ-720IT-E2) (Bulgaria, Germany, Greece, Poland, and Spain) and ActiGraph (Pensacola, FL) accelerometers (Belgium). Step counts from both pedometer and accelerometer are comparable (r = 0.89) and are validated to measure PA in preschool children against the Actigraph accelerometer counts (pedometer: r = 0.64; accelerometer: r = 0.89) [27]. The devices were worn on the right hip, secured by an elastic waist band.

Preschoolers wore the motion sensors for six consecutive days, including two weekend days. Preschoolers’ parents/caregivers received an informational letter and were instructed to let their child wear the measurement devices during all waking hours and to remove the device during water-based activities. After data collection, pedometers were downloaded using Omron Health Management Software version E1.012, and accelerometers were downloaded using ActiLife version 5.5.5-software. Both the first (fitting day) and sixth day (collection day) were omitted, because these days did not have a full day of data and were therefore incomplete. Preschoolers’ step count data were included in data analyses when they had valid data for a minimum of two weekdays and one weekend day. All step counts below 1,000 and above 30,000 steps per day were deleted and treated as missing data [28]. Steps per day were separately calculated for weekdays and weekend days. Steps per weekday and steps per weekend day were dichotomized into 0 (< 11,500 steps/day) and 1 (≥ 11,500 steps/day), to calculate the percentage of preschoolers meeting the PA guideline of 11,500 steps/day which corresponds to 180 minutes of total PA per day [16].

#### Sedentary behaviour

SB (TV viewing, computer use, and quiet play) was assessed by three questions in the core questionnaire. These questions assessed the amount of hours per weekday and per weekend day the child watches TV (ICC_week_ = 0.67; ICC_weekend_ = 0.67), plays on the computer or game consoles (ICC_week_ = 0.72; ICC_weekend_ = 0.81), and plays quietly during leisure time (e.g., drawing, colouring, playing with blocks/puppets; ICC_week_ = 0.42; ICC_weekend_ = 0.50). Answers ranged from”never” to”more than 8 hours per day”, on a nine-point scale. The answer possibilities for these questions are depicted in Table 1. The variables were then recoded into min/day to ensure that numerical outcomes could be used to describe the prevalence of TV viewing, computer use and quiet play. To calculate the percentage of preschoolers meeting the screen time guideline of less than one hour of screen time per day, minutes of TV viewing and computer use were added up and were then dichotomized into 0 (< 60 minutes of screen time per day) and 1 (≥ 60 minutes of screen time per day) [17].

**Table 1:** Questions and answer possibilities on preschoolers’ sedentary behaviour.

	Question	Answer possibilities
Television viewing	About how many hours a day does your child usually watch television (including DVDs and videos) in his/her free time?^1^	1. Never
		2. Less than 30 minutes/day
		3. 30 minutes to <1 hr/day
		4. 1–2 hrs/ day
		5. 3–4 hrs/ day
		6. 5–6 hrs/ day
		7. 7–8 hrs/ day
		8. 8 hrs/ day
		9. More than 8 hrs/ day
Computer use	About how many hours a day does your child use the computer for activities like playing games on a computer, game consoles (e.g., Playstation, Xbox, GameCube) during leisure time?^1^	1. Never
		2. Less than 30 minutes/day
		3. 30 minutes to <1 hr/day
		4. 1–2 hrs/ day
		5. 3–4 hrs/ day
		6. 5–6 hrs/ day
		7. 7–8 hrs/ day
		8. 8 hrs/ day
		9. More than 8 hrs/ day
Quiet play	About how many hours a day does your child have quiet play ((looking into books, playing with blocks, playing with dolls, drawing, construction) during leisure time?^1^	1. Never
		2. Less than 30 minutes/day
		3. 30 minutes to <1 hr/day
		4. 1–2 hrs/ day
		5. 3–4 hrs/ day
		6. 5–6 hrs/ day
		7. 7–8 hrs/ day
		8. 8 hrs/ day
		9. More than 8 hrs/ day

^1^ Separate questions for weekdays and weekend days.

#### Beverage consumption

Beverage consumption (intake of water and intake of sugar-sweetened beverages (SSBs)) was assessed in the FFQ. First, parents/caregivers were asked on how many days per week their child drinks water or soft drinks on a six-point scale ranging from “never or less than once a month” to “every day”. Subsequently, they were asked to indicate how much their child drinks on days they consumed water or soft drinks by ticking the average amount per day, ranging from “100ml or less” to “1000ml or more” on an eleven-point scale. The different answer possibilities for the intake of water and SSBs are depicted in Table 2. Colored food photographs of various portion sizes were printed and added to the questionnaire to help parents/caregivers to quantify to the average portion size consumed by their child on the day of consumption. Mean intake in ml per day was calculated from the FFQ by multiplication of number of days per week and amount per day, divided by seven. Water consumption per day was then dichotomized into 0 (< 500ml per day) and 1 (≥ 500ml per day), to calculate the percentage of preschoolers meeting the water consumption guideline of 500ml or more per day [23].

**Table 2:** Questions and answer possibilities on preschoolers’ water, sugar-sweetened beverage and snack consumption.

	How often does your child consume the following products?	What is the average amount per day?
Water	1. Never or less than once per month	1. 100ml or less
	2. 1–3 days per month	2. Between 100 and 200ml
	3. 1 day per week	3. Between 200 and 300ml
	4. 2–4 days per week	4. Between 300 and 400ml
	5. 5–6 days per week	5. Between 400 and 500ml
	6. Every day	6. Between 500 and 600ml
		7. Between 600 and 700ml
		8. Between 700 and 800ml
		9. Between 800 and 900ml
		10. Between 900 and 1000ml
		11. 1000ml or more
Soft drinks	1. Never or less than once per month	1. 100ml or less
	2. 1–3 days per month	2. Between 100 and 200ml
	3. 1 day per week	3. Between 200 and 300ml
	4. 2–4 days per week	4. Between 300 and 400ml
	5. 5–6 days per week	5. Between 400 and 500ml
	6. Every day	6. Between 500 and 600ml
		7. Between 600 and 700ml
		8. Between 700 and 800ml
		9. Between 800 and 900ml
		10. Between 900 and 1000ml
		11. 1000ml or more
Milk-based desserts (e.g., chocolate mousse, ice cream, custard)	1. Never or less than once per month	1. 50g or less
	2. 1–3 days per month	2. Between 50 and 100g
	3. 1 day per week	3. Between 100 and 150g
	4. 2–4 days per week	4. Between 150 and 200g
	5. 5–6 days per week	5. 200g or more
	6. Every day	
Chocolate and candy bars (e.g., plain chocolate bar, chocolate candy bars)	1. Never or less than once per month	1. 25g or less
	2. 1–3 days per month	2. Between 25 and 50g
	3. 1 day per week	3. Between 50 and 75g
	4. 2–4 days per week	4. Between 75 and 100g
	5. 5–6 days per week	5. Between 100 and 125g
	6. Every day	6.125g or more
Sugar-based desserts (e.g., jelly beans, lollipops, hard candies)	1. Never or less than once per month	1. 5g or less
	2. 1–3 days per month	2. Between 5 and 10g
	3. 1 day per week	3. Between 10 and 15g
	4. 2–4 days per week	4. Between 15 and 20g
	5. 5–6 days per week	5. Between 20 and 25g
	6. Every day	6. Between 25 and 30g
		7. Between 30 and 35g
		8. 35g or more
Cakes	1. Never or less than once per month	1. 35g or less
	2. 1–3 days per month	2. Between 35 and 70g
	3. 1 day per week	3. Between 70 and 105g
	4. 2–4 days per week	4. Between 105 and 140g
	5. 5–6 days per week	5. Between 140 and 175g
	6. Every day	6. Between 175 and 210g
		7. Between 210 and 245g
		8. 245g or more
Biscuits	1. Never or less than once per month	1. 15g or less
	2. 1–3 days per month	2. Between 15 and 30g
	3. 1 day per week	3. Between 30 and 45g
	4. 2–4 days per week	4. Between 45 and 60g
	5. 5–6 days per week	5. 60g or more
	6. Every day	
Salty snacks (e.g., potato chips)	1. Never or less than once per month	1. 25g or less
	2. 1–3 days per month	2. Between 25 and 75g
	3. 1 day per week	3. 75g or more
	4. 2–4 days per week	
	5. 5–6 days per week	
	6. Every day	

#### Unhealthy snacking

Intakes of snacks were assessed by the FFQ. Intakes of milk-based desserts (e.g., chocolate mousse, ice cream, custard), chocolate and candy bars (e.g., plain chocolate bars, chocolate candy bars), sugar-based desserts (e.g., hard candies, jelly beans, lollipops), cakes, biscuits and salty snacks (e.g., potato chips) were each assessed with two food-frequency questions. First, parents/caregivers were asked on how many days per week their child consumed a snack on a six-point scale ranging from “never or less than once a month” to “every day”. Subsequently they were asked to indicate how much their child ate on days they consumed the snack. Parents/caregivers were asked to tick the average amount per day for each of the unhealthy snacking categories. The possible answer categories are depicted in Table 2. Mean intakes in g per day were calculated from the FFQ by multiplication of number of days per week and amount per day in g, divided by seven. Afterwards, mean daily intakes from all six food groups (milk-based desserts, chocolate and candy bars, sugar-based desserts, cakes, biscuits, and salty snacks) were added up and reflected the mean daily intake of snacks in g/day. The validity of the FFQ is currently the aim of another paper in preparation, but preliminary evidence suggests acceptable FFQ validity, with the 3-day-food diary used as the reference method. The FFQ performed better for some items than others. For example, Pearson’s correlation coefficient was 0.30 and 0.34 for biscuits and sugar-based desserts, and 0.24 for water, but did not perform as well for other items such as soft drinks and chocolate, where low or no correlations were observed. This is most likely due to the high level of non-consumers.

### Statistical analyses

Prior to all analyses, all outcome measures were first checked for normal distribution (skewness < 0.70). Sample characteristics were described using SPSS statistics version 20.0 (SPSS Inc, Chicago, IL). To examine whether EBRBs in preschoolers varied across countries, multilevel analyses were performed using MlwiN 2.28 (Centre for Multilevel Modelling, University of Bristol, UK). Multilevel modeling (three-level: child; class; kindergarten) was used to take clustering of children in classes in kindergartens into account. The likelihood ratio test was used to justify that the data fits the model, and analyses were conducted to indicate how much each higher level variable contributed to the variance of the dependent variable. All analyses were adjusted for sex and age. To compare the preschool children who had valid step count data with the preschool children who did not have valid step count data, attrition analyses were conducted as a three-level logistic regression analysis (child; class; kindergarten). For all analyses, statistical significance level was set at p < 0.05. Values are reported as mean and standard error (SE).

## Results

In total, 10,632 preschoolers’ parents/caregivers from six European countries provided written informed consent to participate in the ToyBox cross-sectional study. From those 10,632 preschoolers, step counts were collected in 5,444 preschool children (51.2%), from which 4,045 (38.1%) provided valid data for a minimum of two weekdays and one weekend day (4.77 ± 0.43 years, 52.2% boys). In addition, 8,117 parents/caregivers (76.3%) filled in the core questionnaire (4.78 ± 0.46 years old, 53.0% boys), and 7,244 parents/caregivers (68.1%) filled in the FFQ (4.77 ± 0.44 years old, 52.0% boys). Table 3 depicts the explained variance for each dependent variable at each level of the multilevel model. The between child variance appeared to be significant for all dependent variables (p < 0.001), and the between class variance and the between kindergarten variance was significant for most dependent variables as well. Attrition analyses, comparing 4,045 preschool children with complete pedometer data to those with incomplete pedometer data (n = 1,399), showed no significant differences in age and sex.

**Table 3:** Explained variance for each dependent variable at each level of the multilevel model.

	Level 1: Preschool child			Level 2: Kindergarten class			Level 3: Kindergarten		
	Variance	χ²	p-value	Variance	χ²	p-value	Variance	χ²	p-value
Steps per weekday	63.8%	68.53	**< 0.001**	8.4%	0.58	0.48	27.8%	11.61	**< 0.001**
Steps per weekend day	74.0%	71.67	**< 0.001**	14.0%	1.28	0.26	12.0%	1.79	0.18
PA guidelines weekday	57.1%	45.20	**< 0.001**	8.5%	0.48	0.49	34.4%	16.22	**< 0.001**
PA guidelines weekend day	50.0%	42.35	**< 0.001**	30.1%	5.69	**0.02**	19.9%	4.03	**0.045**
TV weekday	53.0%	82.84	**< 0.001**	21.2%	5.16	**0.02**	25.8%	12.25	**< 0.001**
TV weekend day	38.1%	76.89	**< 0.001**	45.7%	25.65	**< 0.001**	16.2	4.20	**0.04**
Computer weekday	32.1%	75.15	**< 0.001**	22.1%	10.17	**0.001**	45.8%	58.59	**< 0.001**
Computer weekend day	26.1%	74.13	**< 0.001**	30.1%	19.87	**< 0.001**	43.8%	50.69	**< 0.001**
Quiet play weekday	42.5%	152.99	**< 0.001**	0%	/	/	57.5%	240.19	**< 0.001**
Quiet play weekend day	57.1%	82.84	**< 0.001**	1.0%	0	/	41.9%	46.78	**< 0.001**
SB guidelines weekday	57.1%	86.17	**< 0.001**	21.0%	4.74	**0.03**	21.9%	8.42	**0.004**
SB guidelines weekend day	49.7%	81.91	**< 0.001**	30.1%	10.21	**0.001**	20.2%	7.21	**0.007**
Water consumption	50.1%	56.14	**< 0.001**	20.6%	3.98	**0.05**	29.3%	13.72	**< 0.001**
SSB consumption	38.8%	54.95	**< 0.001**	60.0%	33.81	**< 0.001**	1.2%	0.02	0.89
Water guidelines	51.7%	88.31	**< 0.001**	24.5%	7.63	**0.006**	23.8%	11.40	**< 0.001**
Snacking	51.4%	56.42	**< 0.001**	26.2%	5.89	**0.02**	22.4%	7.15	**0.008**

PA = Physical Activity; TV = Television; SB = Sedentary Behaviour; SSB = Sugar-sweetened beverage.

### Physical activity

**Fig 1:**
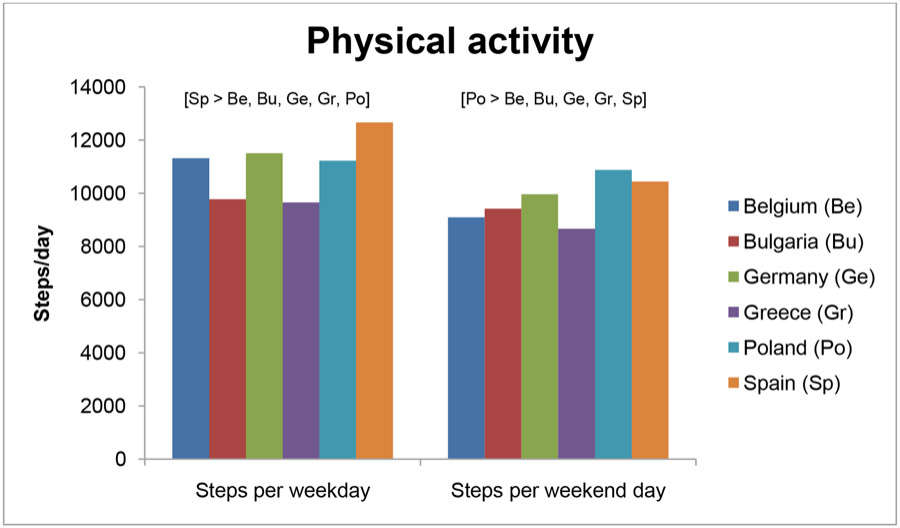
Differences in preschoolers’ physical activity across countries.

The differences in preschoolers’ PA levels across countries are depicted in Table 4 and Fig. 1. Significant differences between countries were found for steps per weekday and steps per weekend day. Greek (9,656 steps/day; SE = 148) and Bulgarian (9,777 steps/day; SE = 154) preschoolers took the least amount of steps per weekday (p<0.001), and Spanish preschool children took the most steps per weekday (12,669 steps/day; SE = 142; p<0.001). For weekend days, steps ranged from 8,667 (SE = 181) steps per weekend day in Greek preschoolers to 10,880 (SE = 125) steps per weekend day for Polish preschool children. Preschoolers meeting the PA guideline of 11,500 steps/day on a weekday were the highest in Spain (60.7%; p<0.001) and the lowest in Greece (26.5%), and Bulgaria (29.3%) (p<0.001). On weekend days, Poland (41.8%) had the highest proportion of preschoolers meeting the PA guidelines (p<0.05), and Greece (20.3%) and Belgium (20.5%) had the lowest proportion of preschool children meeting the PA guidelines of 11,500 steps/day (p<0.01).

**Table 4:** Levels of physical activity in preschool children from six European countries (n = 4,045) (adjusted for sex and age).

	Belgium (n = 806)	Bulgaria (n = 470)	Germany (n = 449)	Greece (n = 575)	Poland (n = 1,192)	Spain (n = 553)
	Steps per day (mean (SE))					
Weekday	11,318 (126)^c^ ^,^ ^e^ ^,^ ^g^	9,777 (154)^b^ ^,^ ^d^ ^,^ ^f^ ^,^ ^g^	11,507 (158)^c^ ^,^ ^e^ ^,^ ^g^	9,656 (148)^b^ ^,^ ^d^ ^,^ ^f^ ^,^ ^g^	11,230 (102)^c^ ^,^ ^e^ ^,^ ^g^	12,669 (142)^a^
Weekend day	9,095 (155)^d^ ^,^ ^f^ ^,^ ^g^	9,426 (194)^d^ ^,^ ^e^ ^,^ ^f^ ^,^ ^g^	9,966 (206)^b^ ^,^ ^c^ ^,^ ^e^ ^,^ ^f^	8,667 (181)^c^ ^,^ ^d^ ^,^ ^f^ ^,^ ^g^	10,880 (125) ^a^	10,438 (173)^b^ ^,^ ^c^ ^,^ ^e^ ^,^ ^f^
	Meeting PA guidelines^1^ (%)					
Weekday	40.0^c^ ^,^ ^d^ ^,^ ^e^ ^,^ ^g^	29.3^b^ ^,^ ^d^ ^,^ ^f^ ^,^ ^g^	49.9^a^	26.5^b^ ^,^ ^d^ ^,^ ^f^ ^,^ ^g^	43.2^c^ ^,^ ^d^ ^,^ ^e^ ^,^ ^g^	60.7^a^
Weekend day	20.5^c^ ^,^ ^d^ ^,^ ^f^ ^,^ ^g^	29.2^b^ ^,^ ^e^ ^,^ ^f^ ^,^ ^g^	31.4^b^ ^,^ ^e^ ^,^ ^f^ ^,^ ^g^	20.3^c^ ^,^ ^d^ ^,^ ^f^ ^,^ ^g^	41.8^a^	37.0^a^

PA = Physical Activity; SE = Standard Error;

^a^ significantly different from the other countries;

^b^ significantly different Belgium;

^c^ significantly different from Bulgaria;

^d^ significantly different from Germany;

^e^ significantly different from Greece;

^f^ significantly different from Poland;

^g^ significantly different from Spain;

^1^ ≥ 11,500 steps per day [16].

### Sedentary behaviour

**Fig 2:**
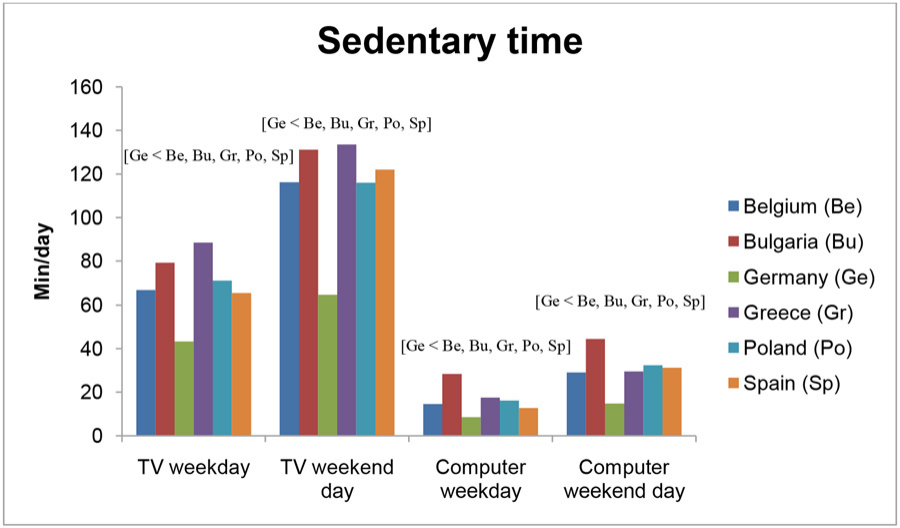
Differences in preschoolers’ sedentary time across countries. TV = Television.

The differences in preschool children’s SB across countries are shown in Table 5 and Fig. 2. German preschoolers had the lowest amount of time watching TV on weekdays (43.3 min/day; SE = 1.5; p<0.001), while Greek preschool children spending the most time watching TV on weekdays (88.5 min/day; SE = 1.4; p<0.001). On weekend days, again German preschoolers had the lowest amount of time watching TV (64.8 min/day; SE = 2.3; p<0.001), while Bulgarian (131.1 min/day; SE = 2.7) and Greek preschoolers (133.5 min/day; SE = 2.0) spent the most time watching TV on weekend days (p<0.05). For computer use on week and weekend days, German preschoolers had the lowest amount of time spent using the computer (8.5 (SE = 0.8) and 14.8 (SE = 1.2) min/day, respectively; p<0.001), while Bulgarian preschool children spent the most time using the computer (28.3 (SE = 0.9) and 44.4 (SE = 1.5) min/day, respectively; p<0.001). Preschoolers’ time spent in quiet play ranged from 60.4 (SE = 2.4) min/day in Spain to 104.9 (SE = 2.0) min/day in Poland for weekdays, and from 95.3 (SE = 3.1) min/day in Bulgaria to 147.2 (SE = 2.8) min/day in Belgium for weekend days. During weekdays, 71.4% of German preschoolers met the screen time guideline of spending less than one hour of screen time per day which was the highest percentage among all countries (p<0.001). Only 24.9% of Bulgarian preschoolers met this guideline, which was the lowest percentage among all countries (p<0.05). During weekend days, this percentage decreased to 52.1% of German preschoolers meeting the screen time guideline, and only 9.2% of Bulgarian preschoolers.

**Table 5:** Levels of sedentary behaviour in preschool children from six European countries (n = 8,117) (adjusted for sex and age).

	Belgium (n = 1,373)	Bulgaria (n = 979)	Germany (n = 1,423)	Greece (n = 1,854)	Poland (n = 1,597)	Spain (n = 891)
	Television viewing (minutes) (mean (SE))					
Weekday	66.9 (1.6)^c^ ^,^ ^d^ ^,^ ^e^ ^,^ ^f^	79.2 (1.8)^a^	43.3 (1.5)^a^	88.5 (1.4)^a^	71.3 (1.5)^a^	65.6 (1.8)^c^ ^,^ ^d^ ^,^ ^e^ ^,^ ^f^
Weekend day	116.2 (2.4)^c^ ^,^ ^d^ ^,^ ^e^	131.1 (2.7)^b^ ^,^ ^d^ ^,^ ^f^ ^,^ ^g^	64.8 (2.3)^a^	133.5 (2.0)^b^ ^,^ ^d^ ^,^ ^f^ ^,^ ^g^	116.0 (2.3)^c^ ^,^ ^d^ ^,^ ^e^	122.0 (2.8)^c^ ^,^ ^d^ ^,^ ^e^
	Computer use (minutes) (mean (SE))					
Weekday	14.6 (0.8)^c^ ^,^ ^d^ ^,^ ^e^	28.3 (0.9)^a^	8.5 (0.8)^a^	17.5 (0.7)^b^ ^,^ ^c^ ^,^ ^d^ ^,^ ^g^	16.1 (0.8)^c^ ^,^ ^d^ ^,^ ^g^	12.7 (1.0)^c^ ^,^ ^d^ ^,^ ^e^ ^,^ ^f^
Weekend day	29.0 (1.3)^c^ ^,^ ^d^ ^,^ ^f^	44.4 (1.5)^a^	14.8 (1.2)^a^	29.5 (1.1)^c^ ^,^ ^d^	32.4 (1.2)^b^ ^,^ ^c^ ^,^ ^d^	31.2 (1.5)^c^ ^,^ ^d^
	Quiet play (minutes) (mean (SE))					
Weekday	70.2 (2.1)^d^ ^,^ ^e^ ^,^ ^f^ ^,^ ^g^	67.8 (2.3)^d^ ^,^ ^e^ ^,^ ^f^ ^,^ ^g^	92.6 (1.9)^a^	86.0 (1.7)^a^	104.9 (2.0)^a^	60.4 (2.4)^a^
Weekend day	147.2 (2.8)^c^ ^,^ ^d^ ^,^ ^e^ ^,^ ^g^	95.3 (3.1)^b^ ^,^ ^d^ ^,^ ^e^ ^,^ ^f^	115.5 (2.6)^b^ ^,^ ^c^ ^,^ ^f^ ^,^ ^g^	114.8 (2.3)^b^ ^,^ ^c^ ^,^ ^f^ ^,^ ^g^	145.6 (2.6)^c^ ^,^ ^d^ ^,^ ^e^ ^,^ ^g^	102.1 (3.2)^b^ ^,^ ^d^ ^,^ ^e^ ^,^ ^f^
	Meeting screen time guidelines^1^ (%)					
Weekday	43.3^c^ ^,^ ^d^ ^,^ ^e^ ^,^ ^f^	24.9^a^	71.4^a^	28.7^a^	37.1^a^	43.9^c^ ^,^ ^d^ ^,^ ^e^ ^,^ ^f^
Weekend day	15.8^c^ ^,^ ^d^ ^,^ ^e^ ^,^ ^g^	9.2^b^ ^,^ ^d^ ^,^ ^f^	52.1^a^	11.8^b^ ^,^ ^d^ ^,^ ^f^	16.1^c^ ^,^ ^d^ ^,^ ^e^ ^,^ ^g^	12.3^b^ ^,^ ^d^ ^,^ ^f^

SB = Sedentary Behaviour; SE = Standard Error;

^a^ significantly different from the other countries;

^b^ significantly different Belgium;

^c^ significantly different from Bulgaria;

^d^ significantly different from Germany;

^e^ significantly different from Greece;

^f^ significantly different from Poland;

^g^ significantly different from Spain;

^1^ limit screen time to less than one hour per day [17].

### Consumption of water, sugar-sweetened beverages, and unhealthy snacks

**Fig 3:**
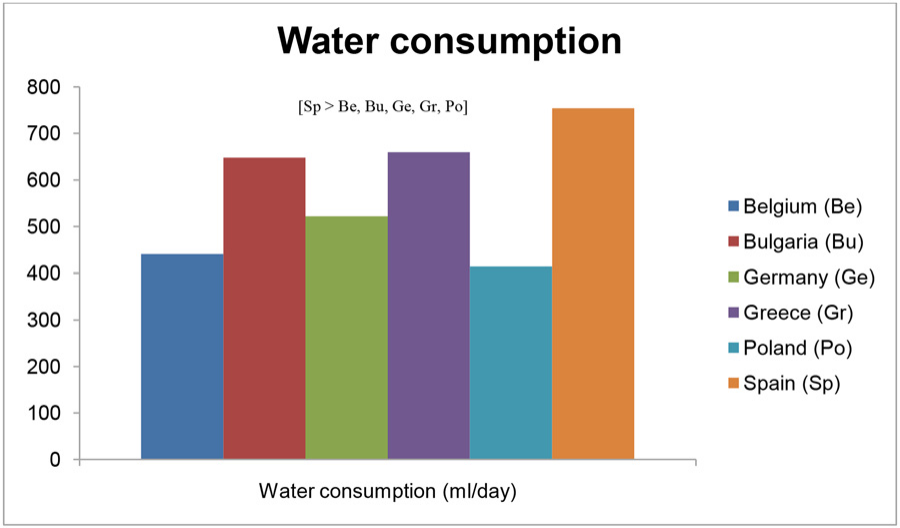
Differences in preschoolers’ water consumption across countries.

**Fig 4:**
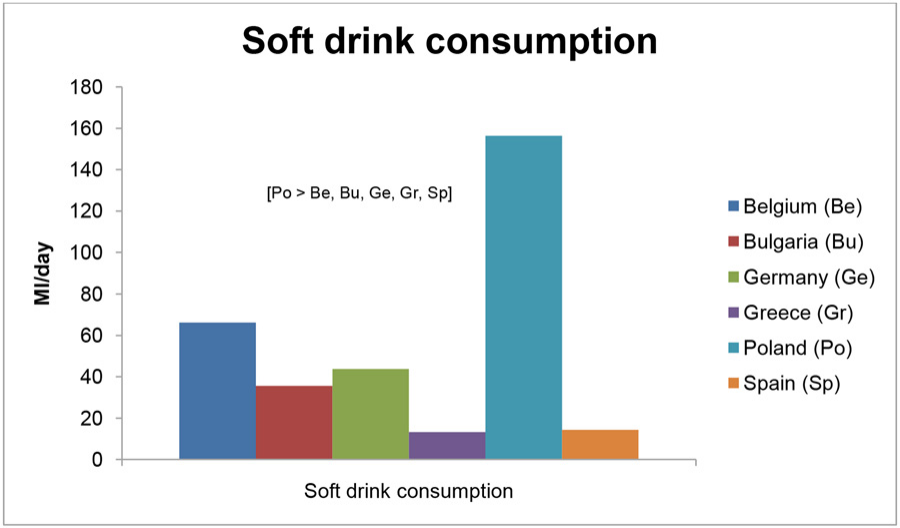
Differences in preschoolers’ soft drink consumption across countries.

The differences in preschool children’s water and SSB consumption and snacking across countries are illustrated in Table 6 and Figs. 3 and 4. The mean water consumption ranged from 414.0 (SE = 8.7) ml/day in Polish preschoolers to 754.0 (SE = 10.4) ml/day in Spanish preschoolers. Spain had the highest proportion of preschool children who complied with the guideline of minimum 500ml water intake per day (81.6%; p<0.01), while Poland (36.0%) and Belgium (37.2%) had the lowest proportion of preschoolers meeting this guideline (p<0.001). The mean intake of SSBs ranged from 13.2 (SE = 4.0) ml/day in Greek preschoolers to 156.4 (SE = 3.9) ml/day in Polish preschool children. The mean snack consumption in preschoolers ranged from 53.3 (SE = 1.6) g/day in Greek preschool children—which was lower than preschoolers of any other country (p<0.05)—to 73.1 (SE = 1.8) g/day in Belgian preschoolers.

**Table 6:** Levels of water and sugar-sweetened beverage consumption and snacking in preschool children from six European countries (n = 7,244) (adjusted for sex and age).

	Belgium (n = 959)	Bulgaria (n = 756)	Germany (n = 1,273)	Greece (n = 1,812)	Poland (n = 1,566)	Spain (n = 878)
	Water and SSB consumption (mean (SE))					
Water (ml per day)	440.9 (10.5)^a^	684.0 (11.3)^b^ ^,^ ^d^ ^,^ ^f^ ^,^ ^g^	522.1 (9.0)^a^	659.6 (9.1)^b^ ^,^ ^d^ ^,^ ^f^ ^,^ ^g^	414.0 (8.7)^a^	754.0 (10.4)^a^
SSBs (ml per day)	66.2 (4.7)^a^	35.6 (5.1)^b^ ^,^ ^e^ ^,^ ^f^ ^,^ ^g^	43.8 (4.0)^b^ ^,^ ^e^ ^,^ ^f^ ^,^ ^g^	13.2 (4.0)^b^ ^,^ ^c^ ^,^ ^d^ ^,^ ^f^	156.4 (3.9)^a^	14.3 (4.6)^b^ ^,^ ^c^ ^,^ ^d^ ^,^ ^f^
	Meeting guidelines^1^ (%)					
Water consumption	37.2^c^ ^,^ ^d^ ^,^ ^e^ ^,^ ^g^	75.5^b^ ^,^ ^d^ ^,^ ^f^ ^,^ ^g^	51.8^a^	72.5^b^ ^,^ ^d^ ^,^ ^f^ ^,^ ^g^	36.0^c^ ^,^ ^d^ ^,^ ^e^ ^,^ ^g^	81.6^a^
	Unhealthy snacking (mean ± SE)					
Snacks (g per day)^2^	73.1 (1.8)^a^	61.7 (1.9)^b^ ^,^ ^e^ ^,^ ^f^	61.4 (1.5)^b^ ^,^ ^e^ ^,^ ^f^	53.3 (1.6)^a^	68.1 (1.5)^a^	58.8 (1.8)^b^ ^,^ ^e^ ^,^ ^f^

SSBs = Sugar-sweetened beverages; SE = Standard Error;

^a^ significantly different from the other countries;

^b^ significantly different Belgium;

^c^ significantly different from Bulgaria;

^d^ significantly different from Germany;

^e^ significantly different from Greece;

^f^ significantly different from Poland;

^g^ significantly different from Spain;

^1^ ≥ 500ml per day [23];

^2^ Snacks = milk-based desserts, chocolate and candy bars, sugar-based desserts, cakes, biscuits, salty snacks.

## Discussion

The current study provided up-to-date information on EBRBs among a sample of four- to six-year-old preschool children across six different European countries. Across all countries, preschoolers frequently engaged in unhealthy EBRBs, namely lack of PA, high levels of SB and unhealthy intakes of beverages and snacks. These unhealthy behaviours are potential risk factors for becoming overweight and obese [4], and varied markedly across countries. Less than half of European preschoolers met the PA threshold of 11,500 steps per day [16], with Greek and Bulgarian preschoolers engaging in the lowest levels of PA, and Spanish and Polish preschoolers engaging in the highest levels of PA. Strikingly, still a large amount of preschool children in the countries with the highest levels of PA (i.e., Spain and Poland) fail to meet the PA recommendations. Although many countries have already developed national PA policies and action plans regarding PA in preschool children [29], these national policy measures and action plans are based on older PA guidelines and should be further developed and updated towards the most recent PA guidelines, since preschoolers’ low PA levels underline the need for further improvement of these policies. Therefore, these policy measures could specifically state that preschool children should accumulate a minimum of 180 minutes of total PA per day [12–15]. Differences in country-specific kindergarten policies and environments might be a possible explanation for the variations in preschoolers’ PA levels. For example, in Spain and Belgium, kindergartens provide physical education (PE) sessions for about two hours per week, while the other four countries do not [30, 31].

Furthermore, a relatively high percentage (71.4% on weekdays and 52.1% on weekend days) of German preschool children—compared with the other countries—met the guidelines of screen viewing behaviour. Only low proportions of preschoolers from the other European countries met the guidelines of limiting screen viewing behaviour to one hour per day, ranging from 24.9% to 43.9% on weekdays and from 9.2% to 16.1% on weekend days. Time spent using a computer was lower in all countries compared to time spent watching TV. A possible explanation might be that the computer is used for educational purposes rather than for pleasure in this age group, which has been mentioned in previously conducted focus groups with preschoolers’ parents/caregivers [32]. In addition, some preschoolers might have an easier access to the TV compared with the computer, as they need parental/caregivers’ supervision to use the computer which is not the case for watching TV [32, 33]. SB guidelines for preschool children mainly focus on reducing screen time in this age group [12], because screen time—and television viewing in particular—is the most common SB in preschool children, and is frequently used as a proxy marker of overall SB in children [12, 20]. However, overall sedentary time is not just made up of screen time, so it is important to measure preschoolers’ time spent in quiet play as well. Based on the descriptive results from the current study, we can conclude that preschoolers also spent considerable amounts of time in quiet play (e.g., looking into books, puzzling, drawing, tinkering), which was equal to or higher than the amount of time spent in screen viewing behaviours for some countries. Although the time spent in quiet play is important for preschoolers’ cognitive development as it is productive SB [12], some activities could be performed in a more active way, for example by performing these activities by standing up. In addition, preschool children could also break up their sitting time. A study in Australian adults showed that breaking up sedentary time causes positive metabolic effects [34]. However, the effects of breaking up sedentary time in preschool children has been understudied [35], which means that more research is needed to confirm these positive metabolic effects in this young age group. The results show that SB interventions not only should target TV viewing—the most commonly targeted SB in preschool children [36, 37]—but also target total sedentary time which includes TV viewing, screen viewing behaviours as well as other SBs like time spent in quiet play [38]. For example, parents/caregivers and teachers could try to avoid prolonged periods of quiet play by promoting activity breaks in between the activities in quiet play. To our knowledge, only Flanders (Belgium) has a national country-specific policy concerning limiting preschoolers’ time spent in SB, and states that children between one and six years old should limit their time spent in prolonged periods of SB [18]. However, policy makers should be made aware of the importance of limiting time spent in SBs in preschool children. This message should also be spread to kindergarten teachers because recently conducted focus groups showed that kindergarten teachers do not see the need to limit preschoolers’ time sitting down since teachers do not perceive this as a problem [39].

Polish preschoolers had the lowest intakes of water, while Spanish preschoolers had the highest intakes of water. Conversely, the highest intakes of SSBs were found in Polish preschool children, while the lowest SSB intakes were found in Greek and Spanish preschoolers. The consumption of snacks was more or less similar across countries, with the highest snack intake in Belgium and the lowest snack intake in Greece. The difference in water consumption across countries might be explained by the differences in climate. In the South-European countries (i.e., Bulgaria, Greece, and Spain), the outside air temperatures during spring and summer are generally higher compared to the West- and Central-European countries (i.e., Belgium, Germany, and Poland), which might mean that preschoolers have a higher thirst to quench, and therefore they might drink water more easily and willingly. Furthermore, the South-European preschoolers might not perceive the need to consume other beverages (e.g., SSBs) since they already have higher intakes of water. On the other hand, the West- and Central-European preschoolers might compensate their low water consumption by a higher intake of SSBs. Conversely, South-European preschoolers might already be able to understand the health risks of SSBs [40], which could result in drinking less SSBs compared to preschool children from the West- and Central-European countries. General national country-specific policies and action plans regarding food and nutrition exist in most European countries [41, 42]. However, in recently conducted focus groups, kindergarten teachers mentioned that different types of beverages are allowed in European kindergartens, meaning that kindergarten policies can differ between countries [43]. Therefore, policy makers should be informed about the importance of decreasing SSB consumption and increasing water consumption among preschool children, which they could address in their policies.

The descriptive results from the ToyBox cross-sectional study support the view that overweight and obesity prevention interventions should target this young age group, and that all EBRBs should be targeted at the same time and not one behaviour at a time, since the current study shows that levels of all EBRBs appear to be a problem in all six countries (e.g., low levels of PA, high levels of SB, high levels of SSBs and unhealthy snacks), but to a different degree. Furthermore, when we look at all EBRBs simultaneously, it is apparent that Spain and the West- and Central-European countries have higher levels of PA and lower levels of SB compared to the other South-European countries. However, West- and Central-European countries have lower intakes of water and higher intakes of SSBs and snacks compared to the South-European countries. The results from the current study show that more standard, general interventions targeting EBRBs in different European countries without room for country-specific adaptations might be questioned since large differences between European preschoolers’ EBRBs were found in the current study. Therefore, future interventions targeting EBRBs in European preschool children should try to target all EBRBs simultaneously, but country-specific adaptations should be made with more emphasis on increasing PA and decreasing SB in South-European countries, and more emphasis on increasing water consumption and decreasing SSB consumption in West- and Central-European countries. Making room for country-specific adaptations in large cross-European intervention studies might therefore increase the effectiveness of these intervention programs in enhancing preschoolers’ EBRBs.

The most important strength of the current study was the large amount of data on preschoolers’ EBRBs across six different European countries, collected using a standardized data collection protocol. By comparing differences in preschoolers’ EBRBs across six European countries, lifestyle differences were detected. Furthermore, possible cluster effects were taken into account, and PA was objectively assessed. A limitation might be the subjective parental report of SB and beverage and snack consumption, which might lead to a possible bias because of parents/caregivers’ social desirability. In addition, parents/caregivers could have reported their child’s behaviour as quiet play, even if there was an active component present. Furthermore, passive transportation was not studied, although passive transportation is also a context of preschool children’s SB. Another limitation was the relatively large drop-out of pedometer data due to insufficient valid days. The preschool children who dropped out might have been the preschoolers who were less physically active. Finally, for preschool children between 5 and 5.5 years old who were included in this study, we used the guidelines for preschool children under five years of age. However, it should be noted that no specific guidelines for overall SB exist in preschool children, which means that more research is needed in this area.

## Conclusion

Differences in preschoolers’ EBRBs were found across six European countries, with Bulgaria and Greece having lower levels of PA and higher levels of SB and Belgium, Germany and Poland having lower intakes of water and higher intakes of SSBs and snacks. Furthermore, low proportions of preschool children met the guidelines for PA, SB and water intake, suggesting that future interventions should target European preschoolers’ EBRBs simultaneously, with room for local and cultural country-specific adaptations. Country-specific policies and interventions are needed to improve preschoolers’ EBRBs.

## Supporting Information

S1 Dataset Dataset physical activity (steps per day). (TIF)

S2 Dataset Dataset snack consumption. (TIF)

S3 Dataset Dataset water consumption and sugar-sweetened beverage consumption. (TIF)

S4 Dataset Dataset sedentary behaviour. (TIF)

## References

[1] de Onis M, Blossner M, Borghi E (2010) Global prevalence and trends of overweight and obesity among preschool children. Am J Clin Nutr; 92: 1257–1264. 10.3945/ajcn.2010.29786 20861173

[2] van Stralen MM, te Velde SJ, van Nassau F, Brug J, Grammatikaki E, et al (2012) Weight status of European preschool children and associations with family demographics and energy balance-related behaviours: a pooled analysis of six European studies. Obes Rev; 13 Suppl 1: 29–41. 10.1111/j.1467-789X.2011.00959.x 22309063

[3] Hill JO, Wyatt HR, Reed GW, Peters JC (2003) Obesity and the environment: where do we go from here? Science; 299: 853–855. 12574618 10.1126/science.1079857

[4] Kremers SP, de Bruijn GJ, Visscher TL, van Mechelen W, de Vries NK, et al (2006) Environmental influences on energy balance-related behaviors: a dual-process view. Int J Behav Nutr Phys Act; 3: 9 16700907 10.1186/1479-5868-3-9 PMC1481572

[5] Ammerman AS, Ward DS, Benjamin SE, Ball SC, Sommers JK, et al (2007) An intervention to promote healthy weight: Nutrition and Physical Activity Self-Assessment for Child Care (NAP SACC) theory and design. Prev Chronic Dis; 4: A67 17572971 PMC1955393

[6] Reilly JJ, Jackson DM, Montgomery C, Kelly LA, Slater C, et al (2004) Total energy expenditure and physical activity in young Scottish children: mixed longitudinal study. Lancet; 363: 211–212. 14738795 10.1016/s0140-6736(03)15331-7

[7] Kelder SH, Perry CL, Klepp KI, Lytle LL (1994) Longitudinal tracking of adolescent smoking, physical activity, and food choice behaviors. Am J Public Health; 84: 1121–1126. 8017536 10.2105/ajph.84.7.1121 PMC1614729

[8] Jones RA, Hinkley T, Okely AD, Salmon J (2013) Tracking physical activity and sedentary behavior in childhood: a systematic review. Am J Prev Med; 44: 651–658. 10.1016/j.amepre.2013.03.001 23683983

[9] Biddle SJ, Pearson N, Ross GM, Braithwaite R (2010) Tracking of sedentary behaviours of young people: a systematic review. Prev Med; 51: 345–351. 10.1016/j.ypmed.2010.07.018 20682330

[10] Northstone K, Emmett PM (2008) Are dietary patterns stable throughout early and mid-childhood? A birth cohort study. Br J Nutr; 100: 1069–1076. 10.1017/S0007114508968264 18377690 PMC2629612

[11] Mikkila V, Rasanen L, Raitakari OT, Pietinen P, Viikari J (2005) Consistent dietary patterns identified from childhood to adulthood: the cardiovascular risk in Young Finns Study. Br J Nutr; 93: 923–931. 16022763 10.1079/bjn20051418

[12] Department of Health and Ageing (2009) Get up and grow: Healthy eating and physical activity for early childhood. Australian Government.

[13] Department of Health Physical Activity Health Improvement and Protection (2011) Start active, stay active. A report on physical activity for health from the four home countries' chief medical officers.

[14] Institute of Medicine of the National Academies (2011) Early Childhood Obesity Prevention Policies. Washington (DC), US: The National Academies Press. 10.3945/an.111.001347 PMC3262615 22332102

[15] Ministerie van Welzijn Volksgezondheid en Gezin (2012) Naar een evenwichtige voeding en beweging.

[16] De Craemer M, De Decker E, De Bourdeaudhuij I, Verloigne M, Manios Y, et al (2014) The translation of preschoolers' physical activity guidelines into a daily step count target. Journal of Sports Sciences; 18:1–7. 10.1080/02640414.2014.981850 25524541

[17] Australian Department of Health and Aging (2009) Get up and grow: Healthy eating and physical activity for early childhood. In: c. o. Australia, editor editors.

[18] Flemish Government Department Health and Family (2012) Vlaamse consensustekst in verband met evenwichtige voeding en beweging, ten behoeve van zorgverstrekkers. Available: http://www.zorg-en-gezondheid.be/uploadedFiles/NLsite_v2/Gezond_leven_en_milieu/Gezonde_voeding_en_beweging/Eetexpert%20project%20consensustekst%2025-07-2012.pdf.

[19] National Association for Sport and Physical Education (NASPE) (2009) Active Start: A Statement of Physical Activity Guidelines for Children From Birth to Age 5 Oxon Hill, Md., USA.: AAHPERD Publications.

[20] Atkin AJ, Gorely T, Clemes SA, Yates T, Edwardson C, et al (2012) Methods of Measurement in epidemiology: sedentary Behaviour. Int J Epidemiol; 41: 1460–1471. 10.1093/ije/dys118 23045206 PMC3465769

[21] Belgian Health Council (2009) Voedingsaanbevelingen voor België—Herziene versie 2009 (Nutritional Recommendations for Belgium. Revised Version 2009). Brussels.

[22] European Food Information Council (2009) Food-based dietary guidelines in Europe.

[23] Nutrition Information Center (2006) De actieve voedingsdriehoek voor kleuters.

[24] Manios Y, Grammatikaki E, Androutsos O, Chinapaw MJ, Gibson EL, et al (2012) A systematic approach for the development of a kindergarten-based intervention for the prevention of obesity in preschool age children: the ToyBox-study. Obes Rev; 13 Suppl 1: 3–12. 10.1111/j.1467-789X.2011.00974.x 22309061

[25] Janz KF, Levy SM, Burns TL, Torner JC, Willing MC, et al (2002) Fatness, physical activity, and television viewing in children during the adiposity rebound period: The Iowa bone development study. Preventive Medicine; 35: 563–571. 12460524 10.1006/pmed.2002.1113

[26] Bayer O, von Kries R, Strauss A, Mitschek C, Toschke AM, et al (2009) Short- and mid-term effects of a setting based prevention program to reduce obesity risk factors in children: a cluster-randomized trial. Clin Nutr; 28: 122–128. 10.1016/j.clnu.2009.01.001 19303675

[27] De Craemer M, De Decker E, Santos-Lozano A, Verloigne M, De Bourdeaudhuij I, et al. (2014) Validity of the Omron pedometer and the actigraph step count function in preschoolers. J Sci Med Sport. 10.1016/j.jsams.2014.06.001 24994695

[28] Rowe DA, Mahar MI, Raedeke TD, Lore J (2004) Measuring physical activity in children with pedometers: Reliability, reactivity, and replacement of missing data. Pediatr Exerc Sci; 16: 343–354.

[29] World Health Organization (2013) Health topics. Disease prevention. Physical activity. Policy.

[30] De Martelaer K, Cools W, Samaey C, Andries C (2007) De school als bron van mogelijkheden om fysiek actief te zijn in de kleuterfase [The school as a source of opportunities to be physically active in the preschool years] In: Van Looy L., Coninx M. and Lochtman K., editors. Onderwijsonderzoek: redelijk eigenzinnig?! [Educational research: reasonable self-willed?!]. Brussels, Belgium: VUBPRESS; 191–206.

[31] Pühse U, Gerber M (2005) International comparison of physical education: Concepts—Problems—Prospects. Oxford: Meyer & Meyer Sport (UK) Ltd; 588–603.

[32] De Decker E, De Craemer M, De Bourdeaudhuij I, Wijndaele K, Duvinage K, et al (2012) Influencing factors of screen time in preschool children: an exploration of parents' perceptions through focus groups in six European countries. Obesity Reviews; 13: 75–84. 10.1111/j.1467-789X.2011.00961.x 22309066

[33] Hinkley T, Salmon J, Okely AD, Trost SG (2010) Correlates of sedentary behaviours in preschool children: a review. Int J Behav Nutr Phys Act; 7: 66 10.1186/1479-5868-7-66 20825682 PMC2945987

[34] Healy GN, Dunstan DW, Salmon J, Cerin E, Shaw JE, et al (2008) Breaks in sedentary time: beneficial associations with metabolic risk. Diabetes Care; 31: 661–666. 10.2337/dc07-2046 18252901

[35] Alghaeed Z, Reilly JJ, Chastin SF, Martin A, Davies G, et al (2013) The influence of minimum sitting period of the ActivPAL on the measurement of breaks in sitting in young children. PLoS One; 8: e71854 10.1371/journal.pone.0071854 23977163 PMC3743753

[36] Dennison BA, Russo TJ, Burdick PA, Jenkins PL (2004) An intervention to reduce television viewing by preschool children. Arch Pediatr Adolesc Med; 158: 170–176. 14757609 10.1001/archpedi.158.2.170

[37] Epstein LH, Roemmich JN, Robinson JL, Paluch RA, Winiewicz DD, et al (2008) A randomized trial of the effects of reducing television viewing and computer use on body mass index in young children. Arch Pediatr Adolesc Med; 162: 239–245. 10.1001/archpediatrics.2007.45 18316661 PMC2291289

[38] Biddle SJ, O'Connell S, Braithwaite RE (2011) Sedentary behaviour interventions in young people: a meta-analysis. Br J Sports Med; 45: 937–942. 10.1136/bjsports-2011-090205 21807671

[39] De Decker E, De Craemer M, De Bourdeaudhuij I, Wijndaele K, Duvinage K, et al (2013) Influencing factors of sedentary behavior in European preschool settings: an exploration through focus groups with teachers. J Sch Health; 83: 654–661. 10.1111/josh.12078 23879785

[40] Calfas KJ, Sallis JF, Nader PR (1991) The development of scales to measure knowledge and preference for diet and physical activity behavior in 4- to 8-year-old children. J Dev Behav Pediatr; 12: 185–190. 1869623

[41] World Health Organization (2013) Global nutrition policy review. What does it take to scale up nutrition action?

[42] World Health Organization (2012) Global database on the Implementation of Nutrition Action (GINA).

[43] De Craemer M, De Decker E, De Bourdeaudhuij I, Deforche B, Vereecken C, et al (2013) Physical activity and beverage consumption in preschoolers: focus groups with parents and teachers. BMC Public Health; 13: 278 10.1186/1471-2458-13-278 23537117 PMC3627633

