# Supplementary figures and images for: Differences in Energy Balance-Related Behaviours in European Preschool Children: The ToyBox-Study
Source: PLoS One. 2015 Mar 18;10(3):e0118303. doi: 10.1371/journal.pone.0118303 (PMC4364763; doi:10.1371/journal.pone.0118303)

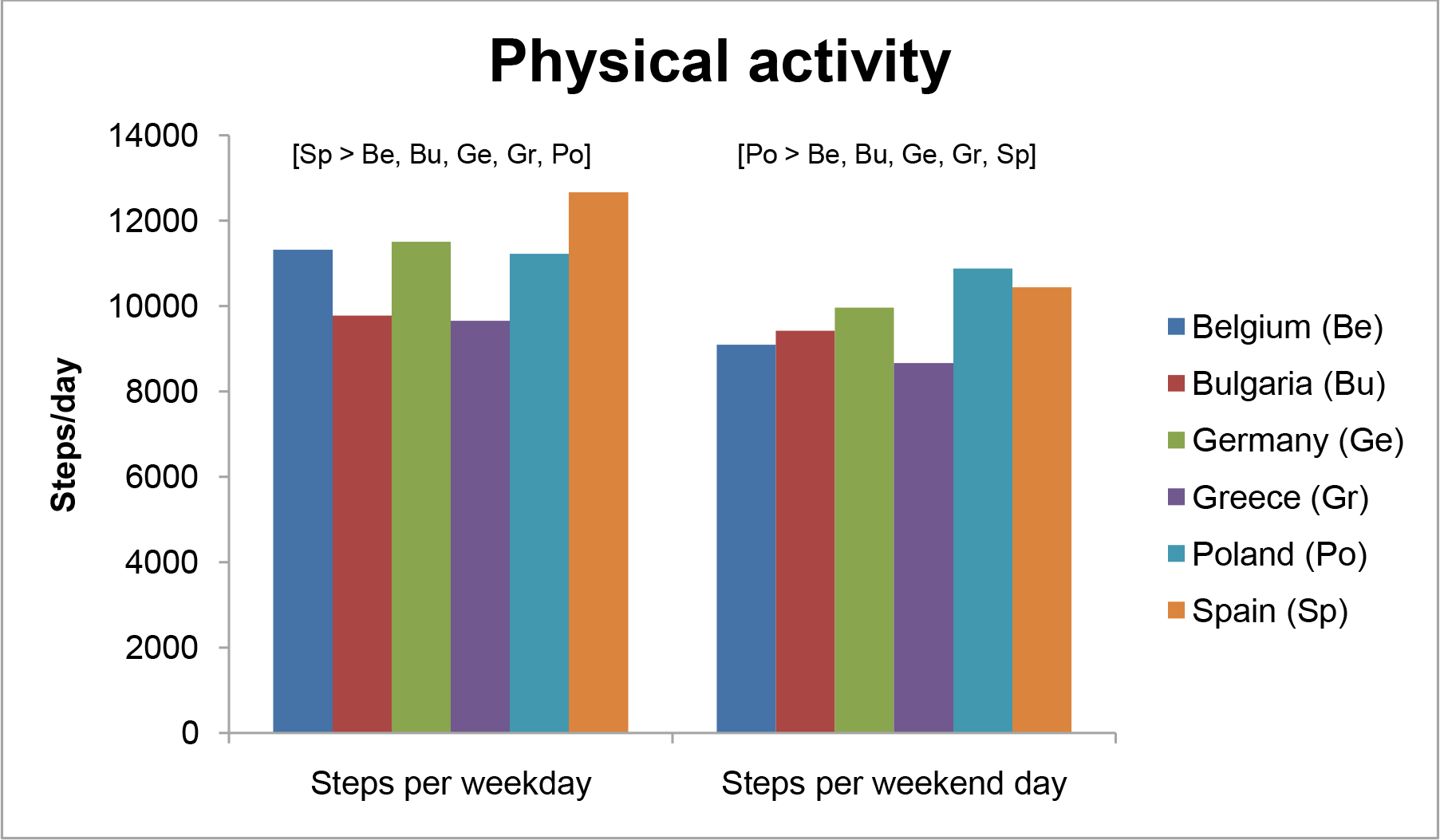

Supplement: S1 Dataset — (TIF) [file pone.0118303.s001.tif]

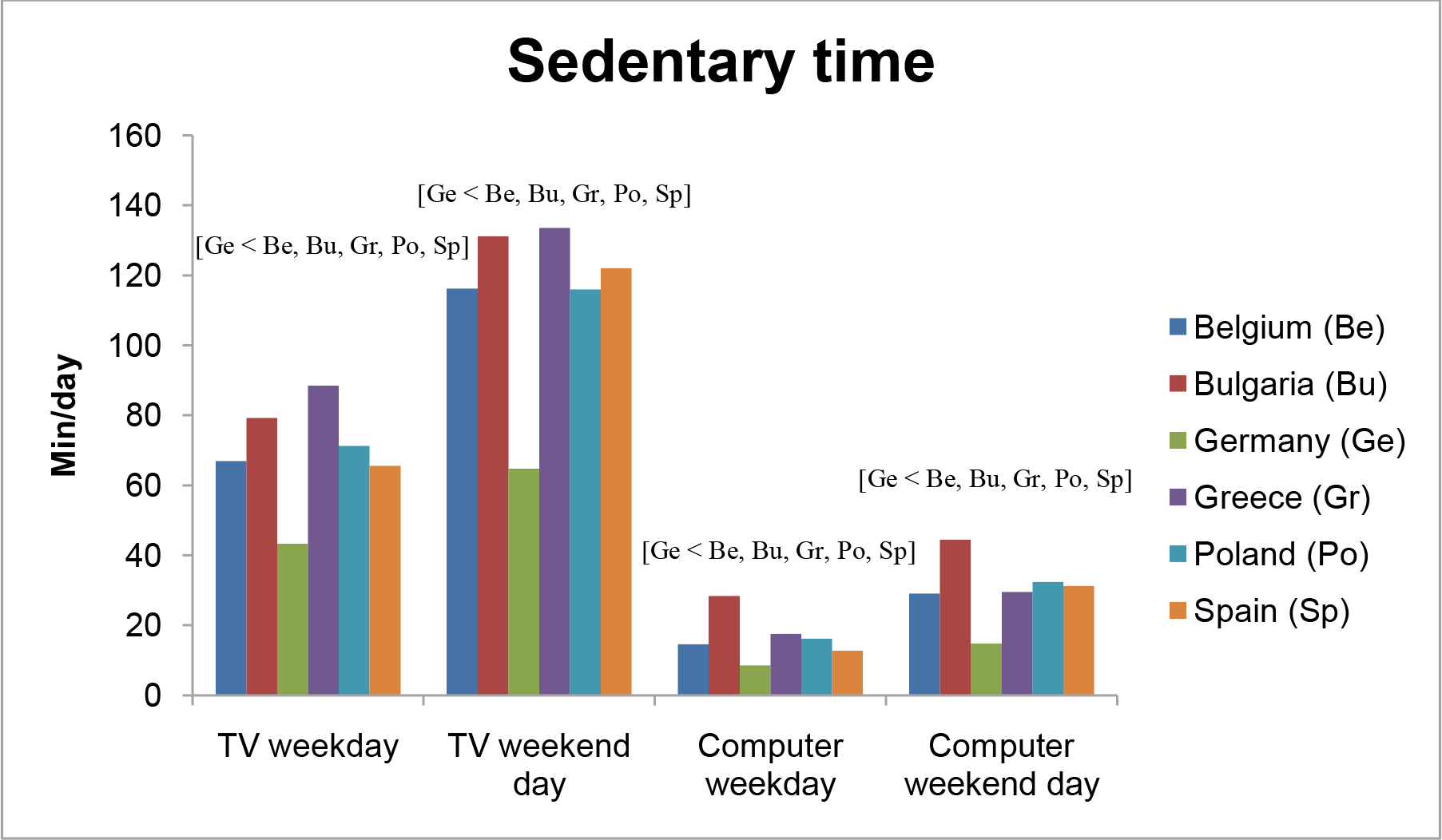

Supplement: S2 Dataset — (TIF) [file pone.0118303.s002.tif]

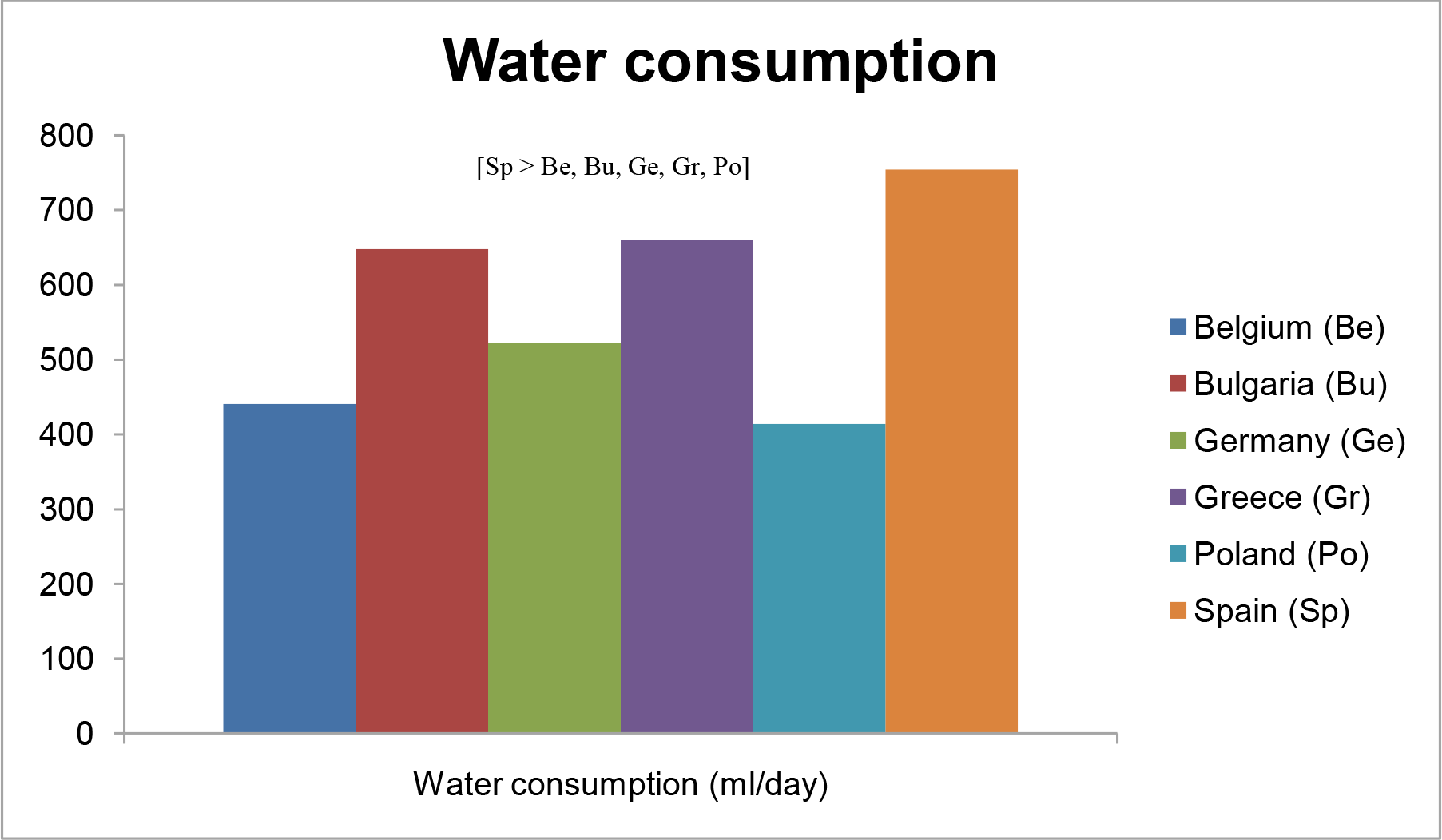

Supplement: S3 Dataset — (TIF) [file pone.0118303.s003.tif]

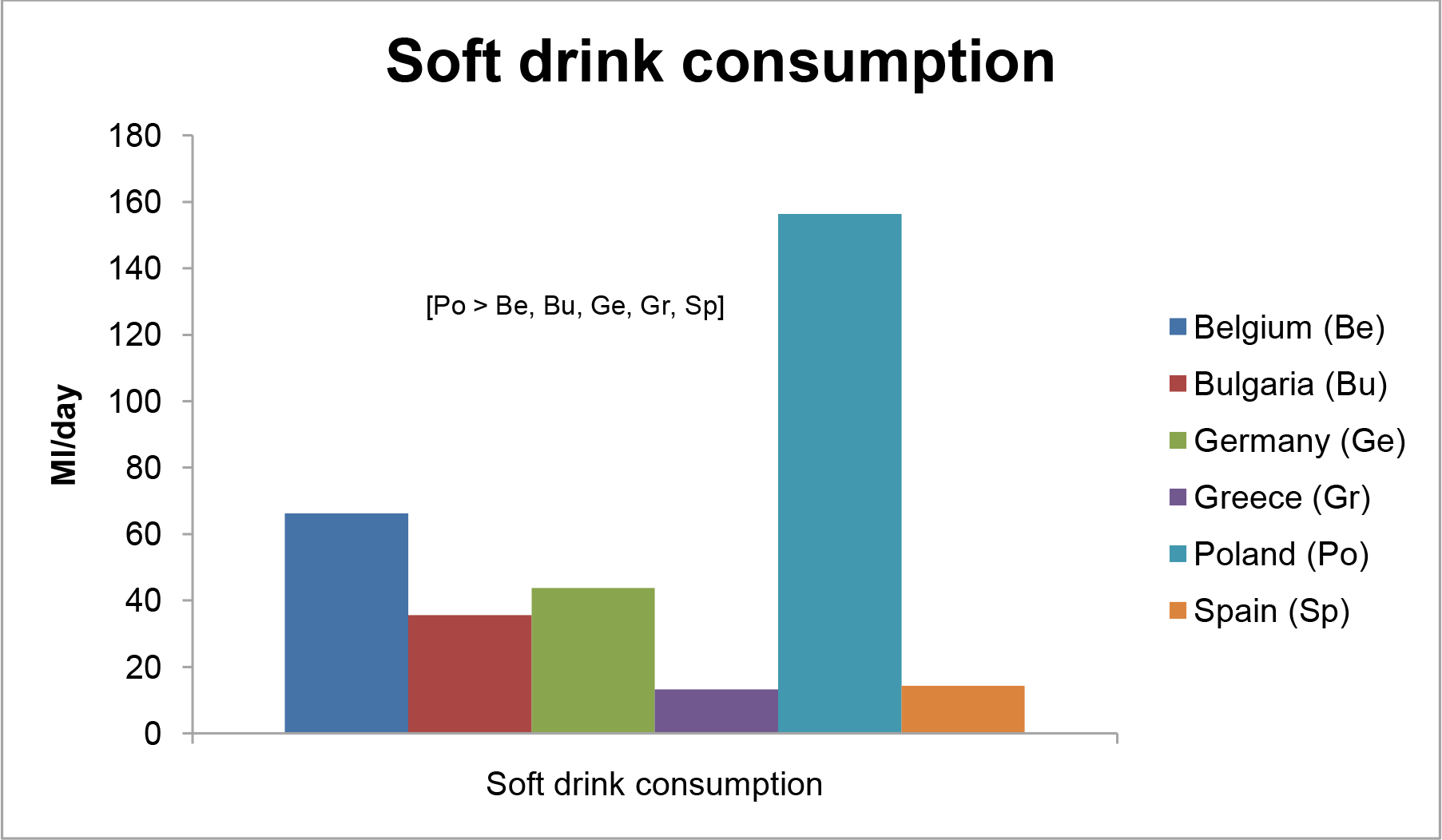

Supplement: S4 Dataset — (TIF) [file pone.0118303.s004.tif]
